# Mapping health care of rare diseases: the example of epidermolysis bullosa in Germany

**DOI:** 10.1186/s13023-018-0944-x

**Published:** 2018-11-08

**Authors:** Antonia Reimer, Leena Bruckner-Tuderman, Hagen Ott

**Affiliations:** 1 Department of Dermatology, Medical Center – University of Freiburg, Hauptstraße 7, 79104 Freiburg, Germany; 2 Department of Paediatric Dermatology, Children’s Hospital AUF DER BULT, Janusz-Korczak-Allee 12, 30173 Hannover, Germany

**Keywords:** Rare disease, Genodermatoses, Epidermolysis bullosa, Health care, Survey, Paediatric dermatology

## Abstract

**Background:**

Rare diseases affect approximately 30 million people in the European Union and present a major health issue. Over 1000 rare skin diseases are known, many of which are of genetic origin and manifest in childhood. One of these diseases is epidermolysis bullosa (EB), a genodermatosis presenting with skin fragility and blistering. With an estimate of up to 2000 affected individuals in Germany, many of these children, but only two specialist centres, the question arose where and how health care for this rare disease is provided. This question was addressed by an online survey of all paediatric and dermatological departments in Germany.

**Results:**

The response rate was 40.5% (203/501), and 39 departments confirmed treating EB (7.8% of the units addressed). Health care for individuals with EB was provided both by dermatological and paediatric departments (19.8 and 4.2% respectively). The geographic distribution of EB health care was uneven. The two EB centres in Hanover and Freiburg treated 70 and 113 patients, two other departments saw 11 to 20 patients, while the majority saw less than 10 patients annually. There existed large variations between 1. the consultation setting, time frame and frequency, 2. the recommended examinations and check-ups and 3. the diagnostics used to establish the diagnosis. Over 50% of participating physicians were dissatisfied with health care outside of hospitals and more than 20% with their patients’ supply with bandages or medications.

**Conclusions:**

The survey results show that health care for individuals with EB in Germany is provided multidisciplinarily. Approaches to diagnostics and follow-up recommendations are heterogeneous and national guidelines are lacking. Functioning and innovative political structures are needed to improve networking and strengthening specialised centres to meet the special needs of individuals with EB and other rare diseases.

## Background

Rare diseases are defined as occurring in less than 5 in 10 000 people, and up to 8000 different rare diseases are assumed to affect approximately 30 million people in the European Union [1]. Rare diseases have been identified as a priority health topic by the European Commission and others [1–4]. Time to diagnosis can be years [5], and experts on the diseases can be as rare as the disease itself. Affected individuals suffer from reduced quality of life [6] and strive to find experienced healthcare professionals for their disease. In 2017, the European reference network (ERN) Skin was founded to coordinate care and research on rare skin diseases, and national action plans have been established (e.g. the German national action plan for rare diseases, NAMSE, in 2013 [7]). Registers for several rare diseases in Germany have evolved over time in institutions that have collected the disease-specific expertise, but these registers are decentral, use different custom-made database systems and are mainly inaccessibly from outside the institutions. It is generally accepted that individuals with rare diseases should primarily be treated in specialised centres, but travel to these centres can be a burden and in between visits, every-day requirements and medical conditions may need to be addressed and treated in the patients’ home area. Germany currently counts 82.5 million inhabitants, thereof 11 million children under the age of 15 years, and is equipped with 385 paediatric and 119 dermatologic departments throughout the country (including small units and rehabilitation clinics). Here, we assessed the share of these departments in the health care of rare skin disease by conducting an online survey using the genodermatosis epidermolysis bullosa (EB) as an example.

As a large group of hereditary diseases, EB is classified into four main subtypes (EB simplex, junctional EB, dystrophic EB and Kindler syndrome), which differ vastly in their clinical presentation and prognosis [8]. The overall incidence of EB in Germany is estimated at 1:25.000 births, with a prevalence of 25–50:1.000.000, currently resulting in up to 2000 affected individuals in Germany [9]. So far, mutations in 20 genes involved in EB have been identified, which lead to dysfunctionality in or complete absence of structural skin proteins. While many molecular mechanisms of EB pathogenesis are understood, and despite very promising recent gene therapy investigations [10], no causal or curative therapy has been reached to date. This presumably leads to different standards of care between health care providers and to insecurity as to which examinations and treatments should be covered by health insurances.

In Germany, two EB centres provide comprehensive clinical care and clinical trial facilities: Freiburg and Hanover, of which Freiburg additionally offers the full spectrum of diagnostics and research. It is unknown whether all individuals with EB present to these centres at all. Due to geographical reasons and long journeys, some patients present after longer intervals than recommended by the centres, so the question arises if and, if yes, where EB-specific health care is provided in the meantime. In many countries, such as the USA or Canada, paediatric dermatologists coordinate care for individuals with EB [11]. As there is no official subspecialty for paediatric dermatology in Germany, both paediatric and dermatologic departments are involved in clinical care for EB in this country. Currently, it is unclear how many and what types of patients are treated at which level of expertise outside the EB centres. We hypothesized that EB health care in Germany is unevenly distributed and that levels of care vary. This study aimed to identify the providers of EB health care in Germany and to assess the standard level of care using an online survey. The new knowledge will facilitate communication and networking in between professionals, allow stratification of centres e.g. for clinical trials, and eventually improve patient care.

## Results

### Participants and hospital characteristics

**Fig. 1:**
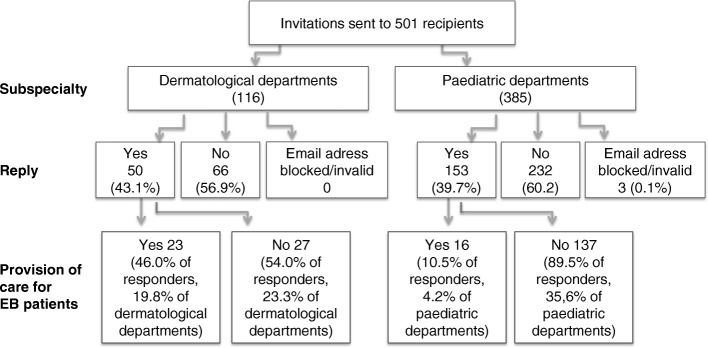
Flowchart of the survey response

The overall survey response rate was 40.5% (203/501); 43.1% (50/116) of dermatological departments and 39.7% (153/385) of paediatric departments participated in the survey (Fig. 1). Most participants were based in community hospitals (29.1%), followed by university hospitals (22.7%), private hospitals (22.7%), confessional (11.3%) and charitable trust hospitals (4.4%; others: 8.9%, NA: 0.9%). EB care was provided by 19.2% participants (39/203; 7.8% of all departments addressed). University hospitals were the main providers of EB care (43.5%).

### Patient numbers and characteristics; geographical distribution and setting of EB care

**Fig. 2:**
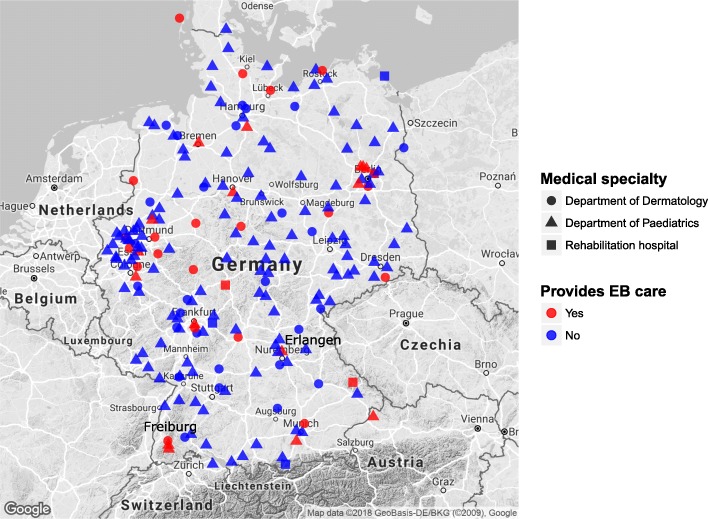
Geographical location of all survey participants according to specialisation of their clinic (Department of Dermatology, Paediatrics or rehabilitation hospitals) and the provision of care for individuals with EB (yes = red, no = blue)

**Fig. 3:**
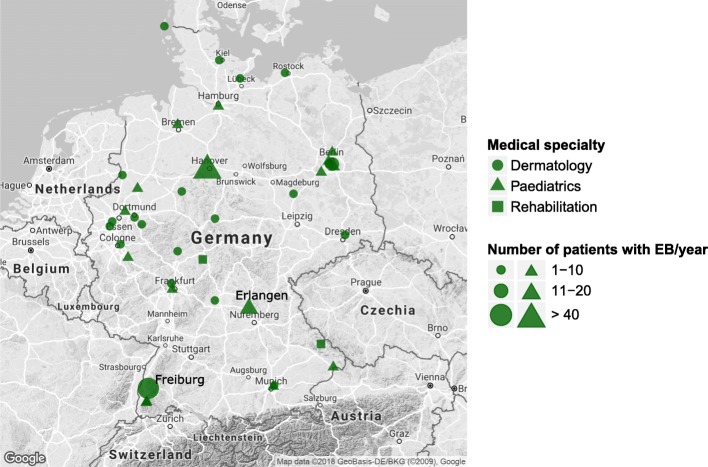
Geographical location of survey participants that confirmed to provide care for individuals with EB. Symbol size indicates the number of patients treated annually, symbol shape indicates the clinics’ specialisations. In some cities, several departments or hospitals are involved in EB care; this is visualised by overlay of symbols. Note that only 2 hospitals indicated the care for > 40 patients each (Freiburg 113, Hanover 70 patients) and 2 for 11–20 patients (Erlangen, Berlin)

Both dermatologic and paediatric departments are involved in EB care (19.8 and 4.2% respectively, comp. Figure 1). The geographical mapping of survey participants shows an uneven distribution of EB-care providers with the majority in north-western Germany (including the highly populated Ruhr area), six in north-eastern and four in both south-eastern and south-western Germany (Fig. 2). As to the number of EB patients treated per hospital, the two German EB centres provided care for most patients. Freiburg saw 113 and Hanover approximately 70 patients in the past 12 months. Both the Department of Paediatrics, University Hospital Erlangen, Erlangen, and the Department of Dermatology and Allergy, Charité-Universitätmedizin Berlin, Berlin treated 11–20 patients annually. All other institutions (35/39, 89.7%) stated annual numbers of 10 or less patients (Fig. 3).

**Fig. 4:**
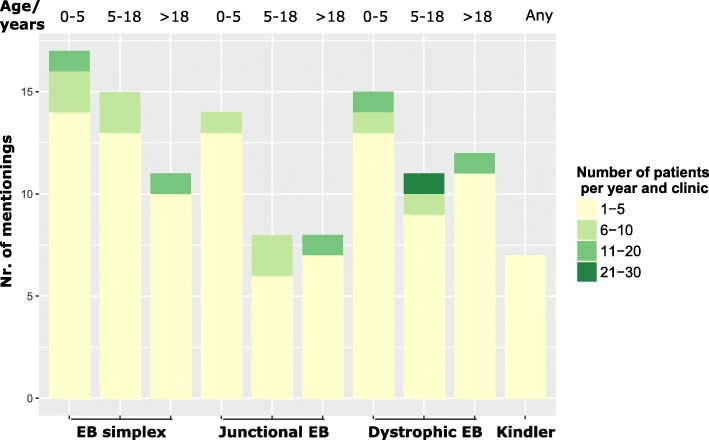
Number of individuals with EB per subtype and age group treated by survey participants during the previous twelve months

Regarding the four main subtypes of EB, EBS and DEB were most frequently encountered by the survey participants, followed by JEB. The clear majority of EB health care providers saw less than 5 patients per subtype and age group (Fig. 4), numbers of 10 or more were only reached by the EB centres Freiburg and Hanover. Seven participants confirmed that they saw patients with Kindler syndrome, but none more than five patients in total.

Most responders caring for EB patients stated that they saw their patients in case of problems (40%, *n* = 22) or did not have a standardized approach on consultation frequency (20%, *n* = 11). Clinical follow-ups every 3 months were recommended by 20% (n = 11), every 6 months by 14.5% (*n* = 8) and every 12 months by 5.5% (*n* = 3). Multiple answers to this question were possible.

Care was mainly provided through an outpatient clinic (56.4% of responses), either in specific consultation hours (33.3%, e.g. for paediatric dermatology, genodermatoses or in one case a specific EB-consultation hour), in outpatient clinics for general dermatology or paediatrics (18.0%) or in an emergency setting (5.1%). In 38.5%, in-patient stay was specified as the main setting for EB care. Two hospitals (i.e., the EB centres Freiburg and Hanover, 5.1%) indicated day care as their main mode of consultation.

**Fig. 5:**
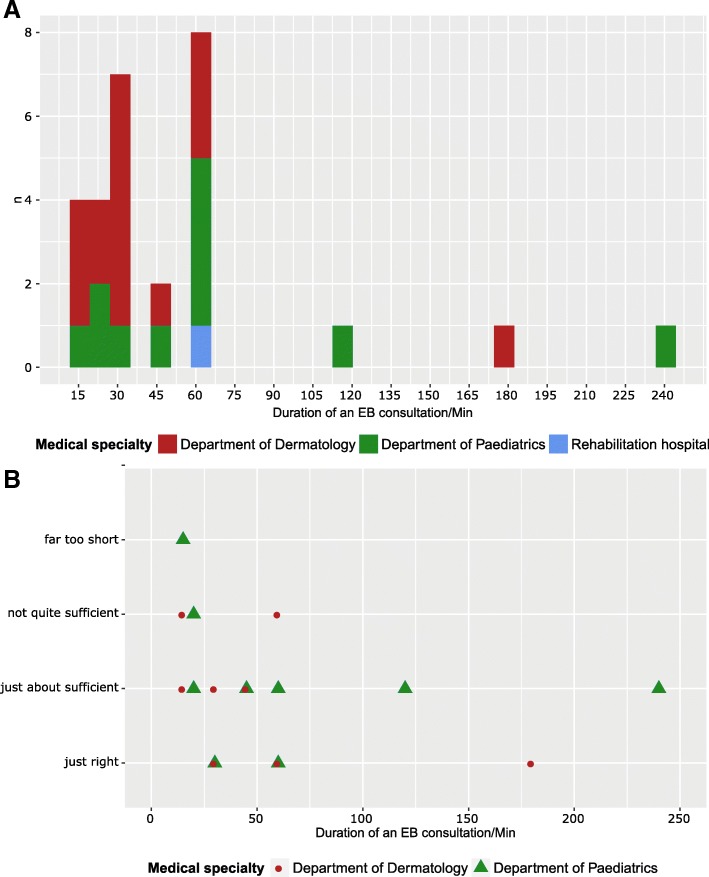
Average time (minutes) available per EB consultation (**a**) and physicians’ satisfaction regarding the average time (minutes) available per EB consultation (**b**). Numerical answers to consultation times were available from 27/39 participants

The average time of an EB consultation was 52.1 min (range 15–240, median 30 min) with a broad variability: While 88.9% of participants specified an average consultation time of 60 min or less per patient, three responders had an average of 120, 180 and 240 min per patient respectively (two of these were the EB centres Freiburg and Hanover, both in a day care setting) (Fig. 5a). There was vast variation amongst physicians regarding their satisfaction with the available time per patient (Fig. 5b).

### Diagnostics of EB

To secure the diagnosis of suspected EB, commonly used methods by the participants comprised the clinical evaluation (25.9%), mutation analysis (23.2%), immunofluorescence mapping (19.6%) and dermatohistopathology (17.9%). Electron microscopy was mentioned by 4 participants (3.6%). In 9.8%, patients presenting to the participating departments had already been diagnosed elsewhere.

### Professionals involved in EB care

**Fig. 6:**
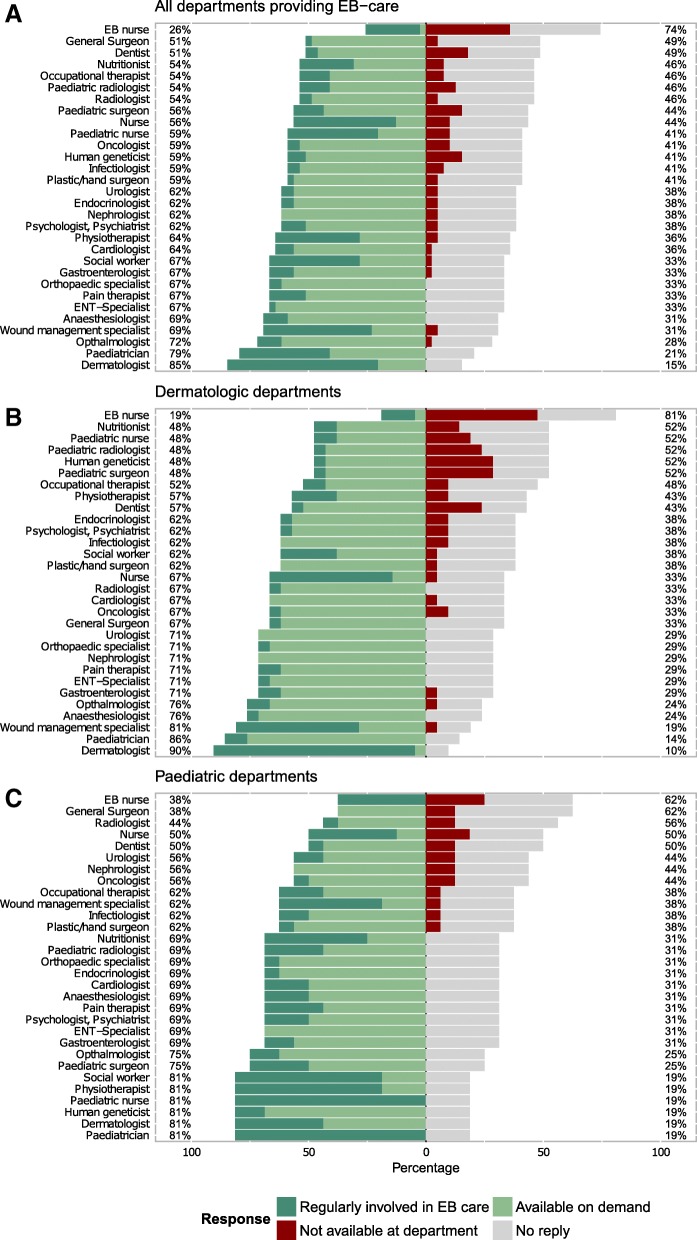
Medical subspecialties involved in EB care. **a** Overall response (*n* = 39). **b** Responses of departments for Dermatology (*n* = 21), **c** Departments for Paediatrics (*n* = 16). Replies on medical subspecialties in rehabilitation hospitals were only available for one of two hospitals, and only a dermatologist was regularly involved in EB care in this hospital

Medical subspecialties involved in EB care included dermatologists, paediatricians and nurses (including paediatric nurses and nurses experienced in EB care). Physiotherapists, nutritionists, wound managers and social workers were also indicated as frequent members of the team involved in EB care. In most departments, the majority of subspecialties were available for consultations (Fig. 6). EB nurses, a professional title not protected/available by any exam in Germany, but rather assigned to credit personal professional experience with EB, were available in 26% of clinics and if available near to all involved in patient care.

### Clinical and laboratory parameters for follow-up of EB patients

**Fig. 7:**
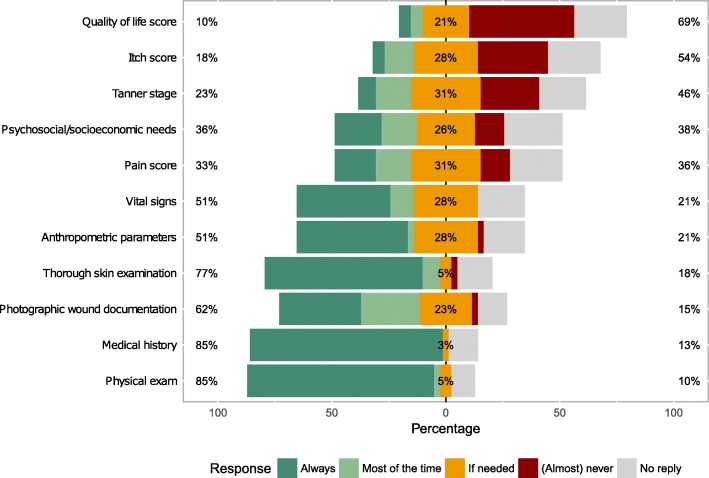
Medical history and clinical parameters collected during visits of EB patients. Anthropometric parameters include weight, height and calculation of percentiles

Measures during visits of EB patients included medical history, physical examination and complete skin (cancer) screening (Fig. 7). Body measurements and measurements of vital parameters were taken in all or most of the visits, otherwise “if needed” (presumably in case of fever or other signs of infections). Photographic documentation was used by 75% of participants, regularly so by 62%. Clinical scores to assess pain were used in 33 to 64% and included visual analogue or numeric rating, KUSS (paediatric scale of discomfort and pain, *Kindliche Unbehagens- und Schmerz-Skala*) and COMFORT scales (collected via free text fields). Itch was assessed regularly by 18% of participants caring for EB, e.g. with visual analogue or numeric rating and Itch-Man scales. Quality of life was assessed in 10 to 31% of cases, the scores used included DLQI (Dermatology Life Quality Index), CDLQI (Children’s Dermatology Life Quality Index) and CHAQ (Child Health Assessment Questionnaire).

**Fig. 8:**
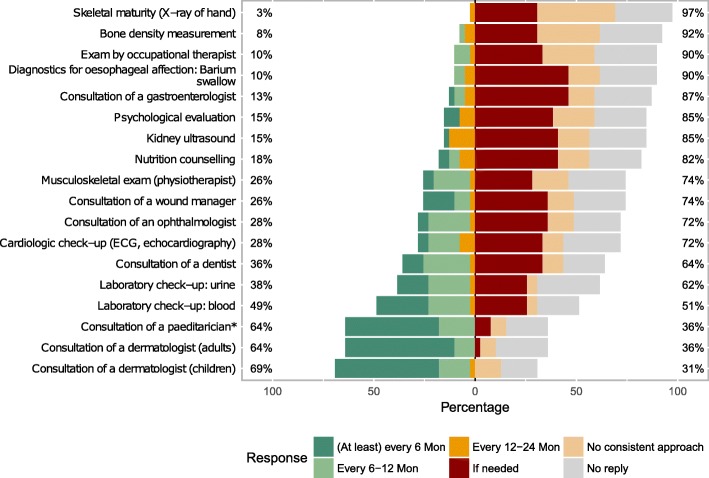
Participants’ recommendations regarding frequency of various examinations in EB-patients with generalised disease. *Additional to paediatric check-ups according to national health plan

Regarding recommendations for check-ups for individuals with generalised EB (primarily junctional and dystrophic subtypes), there was a consensus for regular consultations of dermatologists and paediatricians in intervals of at least every 6 to 6–12 months by two thirds of the participants. Recommendations for laboratory check-ups and instrument-based diagnostics differed considerably (Fig. 8).

With regard to oral health care, there was no difference in the recommendation for the time point of the first examination by a dentist between EB subtypes. Participants recommended a first visit to the dentist right after dentition in 27%, at around 12 months of age in 10.7% and later than that in 1.3%. There was no consistent approach in 40.9% of participants (no reply: 18.2%). During the further course of disease, dentist consultations were recommended at least every 6 months by 10.2%, every 6–12 months by 23.1 and every 12–24 by 2.6%. One third recommended dentist visits “if needed”, 10.3% did not have a consistent approach to the topic.

**Fig. 9:**
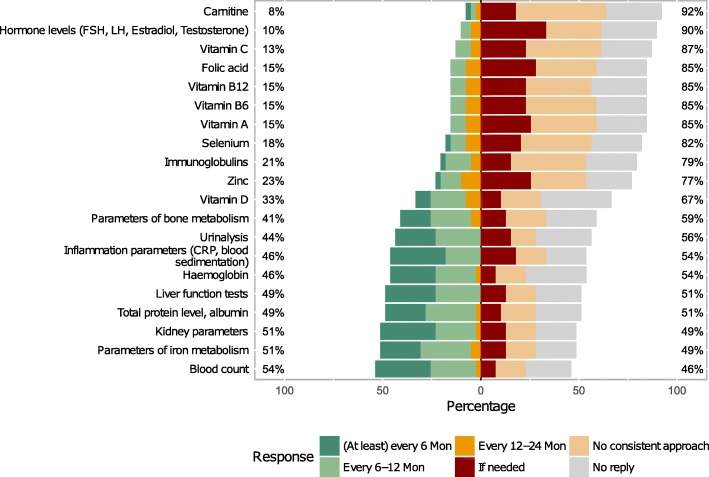
Participants’ practices and recommendations regarding the frequency of specific laboratory measurements

Among the laboratory parameters most frequently checked or recommended were blood count, anaemia parameters (haemoglobin, reticulocytes, ferritin, transferrin saturation and iron levels), parameters of kidney function (urea, creatinine), protein levels, inflammation markers (C-reactive protein, ferritin, calcitonin) and indicators of bone metabolism (calcium, phosphate, alkaline phosphatase, vitamin D). Mostly, there was no consistent approach regarding the measurement of vitamins and micronutrients, although among these, vitamin D and zinc were most frequently analysed (Fig. 9).

Consultations of human geneticists were recommended by nearly all participants (92.3%, no answer 7.7%). Asked for the time point for such a consultation (multiple answers possible), 30.4% participants suggested the time of diagnosis (regardless of age), 19.6% when desired by the patient, 18.5% in case the parents considered another pregnancy, and 16.3% in case the individual with EB him- or herself considered having children. 8.7% did not follow a consistent approach, 3.3% recommended genetic counselling in infancy and 3.3% in adolescence.

Rehabilitation (in Germany mostly performed in inpatient settings) was generally recommended by 41% (*n* = 16) and recommended in some instances by 33.3% (*n* = 13) of participants. Only 7.7% of the participants did not recommend rehabilitation for their patients (no answer in 17.9% (*n* = 7)).

### Participation in clinical trials

Seven participating institutions (17.9%) confirmed to currently participate in clinical trials regarding EB (Bochum, Erlangen, Freiburg (2 departments), Hanover, Kiel, Rostock). They stated a mean number of 1.8 ongoing trials (range 0–5, median 1). Trial participation was negated in 74.4% (*n* = 29), no answer was available in 7.7%.

### Physicians’ perception of EB care outside their hospitals

The perception of care for EB patients outside of hospitals was addressed with four subjective questions. Only 12.8% felt that follow-up care by the patients’ general practitioners, paediatricians and/or dermatologists were rather sufficient, while 33.3% felt it to be rather insufficient and 20.5% not sufficient at all. The recommended follow-up examinations and therapies were, in the participants’ opinion, for the most part implemented in 48.2%, rather not in 15.4% and not at all implemented in 2.6% (*n* = 1). Domestic nursing for EB was stated by 33.3% of participants to essentially meet the demand, by 23.1% to not quite meet the demand and by 5.2% (*n* = 2) to not meet the demand at all. The supply with bandages and medication in follow-up prescriptions was in complete accordance to the participants’ recommendations in 7.7% (*n* = 3), rather in accordance with their recommendations in 35.9%, rather not following recommendations in 20.5% and not at all so in 5.1%.

## Discussion

Patients with potentially life-threatening, rare diseases and their families critically depend upon specialised centres providing both acute treatment in case of emergency and elective multiprofessional follow-up care at regular intervals. EB comprises a large group of different diseases that share skin fragility and blistering [8]. The severity of affection and complications differ vastly between EB subtypes. This entails that a hospital experienced with, for example, EB simplex, may not necessarily be competent or confident to treat patients with generalized junctional or dystrophic EB.

Patient surveys have clearly shown that not even in industrialized countries with high standards of medical care, the requirements of people with rare diseases are met, especially with regard to establishing the correct diagnosis, obtaining comprehensible information and finding high-level clinical expertise [5]. The negative impact on health-related quality of life of the affected individual and the entire family and the high socio-economic burden of EB have been demonstrated [12–15]. Patients with rare diseases are often well connected through support groups and social media [12]. Thus, the authors will make the survey results available to the German EB community via the national patient support group. For individuals with EB in Germany, the maps and results obtained in our study therefore provide a valuable guide to make informed decisions regarding their disease and health care.

The distribution of hospitals treating EB patients in Germany is uneven, as are the diagnostic and therapeutic approaches among these departments. The focus and need of EB consultations varies over the course of disease. During infancy, disease burden is particularly high and parents have a high need for advice and guidance. This is opposed to only 4.2% of paediatric hospitals providing EB care. Although 19.8% of dermatological hospitals provide EB care, infants and young children are traditionally not treated as in-patients in adult dermatology units in Germany. This contributes to a lack of adequate care facilities in the pediatric age group, especially as paediatric dermatology is not an official medical subspecialty in Germany. A special constellation is the occurrence of squamous cell carcinoma in individuals with dystrophic EB from adolescence onwards, the most common cause of premature death in this group [16]. Dermatological hospitals are better trained in the diagnosis and treatment of these aggressive tumors in comparison to paediatric facilities. Our results allow for the identification of interdisciplinary networking partners to consult in complex clinical situations, thus strengthening EB networks such as the German EB network established in 2003 [9].

Personal experience in the EB centres Freiburg and Hannover shows that medical counselling of EB patients and their physicians largely relies on telephone and email. This service is currently provided without any monetary compensation or formal requirements, and thus partly depends on the economic resources and benevolence of the physicians and the respective hospital operators. Moreover, improved facilities for safe data exchange as well as up-to-date telemedicine structures are urgently needed. Politics, health care authorities and insurance companies are requested to provide easily accessible, operable and refundable medical services to implement telemedicine as an integral part of rare disease health care in the near future.

The survey results also demonstrate that diagnostics and follow-up care of EB patients are heterogeneous. Only 23.2% of survey participants confirmed their patients’ diagnosis by mutation analysis. This is surprising as mutation analysis is covered by health insurances in Germany if certain administrative rules are followed. In contrast, 79% of EB cases presenting to the EB center Freiburg are currently confirmed by molecular diagnostics. Knowledge of the mutational background is especially relevant with regard to recent developments of targeted therapies such as exon skipping or gene therapy [10, 17, 18].

A thorough consultation for an EB patient includes time-consuming and potentially painful bandage changes, detailed skin inspection and (photographic) documentation; anesthesia procedures can be necessary. The outpatient care setting chosen in 56.4% of participating departments is very often insufficient in providing this thorough consultation, especially in most cases of severe EB subtypes. These departments were often content with 30 to 60 min of consultation time, suggesting that either they treat less severely affected individuals or may not be aware of the needs of individuals with EB. The EB centers Freiburg and Hannover have implemented day-care settings with 180 to 240 min time per patient. Still, satisfaction with the amount of time is not complete due to the engagement in more severe and often very complex cases. This reflects the depth of engagement and the holistic approach to this rare disease spectrum that are unique for specialised centres. Simultaneously, monetary compensation does not meet the expenses generated by this multi-expert care, time requirements and bandage materials. To provide such a high-standard service in the future, adequate economic compensation, e.g. through disease management programs for rare diseases or other special remuneration, is urgently needed.

Although tangible recommendations and problem-oriented clinical practise guidelines exist, e.g. for the management of wounds [19], oral healthcare [20], laboratory check ups [21] or squamous cell carcinomas in EB [16], authoritative national or international guidelines for general EB care are lacking. Such guidelines have been published in other countries [22] and have recently been established for the treatment of congenital ichthyoses, another severe genodermatosis, both in Germany and on a European level [23, 24]. National guidelines for EB would certainly help to provide structured and consistent care with instant benefit for affected individuals and their caregivers.

The high number of individuals affected by a variety of rare diseases calls for transnational alliances and projects. Recently founded structures (e. g. E-Rare, ERN) and the platform Orphanet established in 1997 are promising instruments. In this context, our study underlines that the reality of medical care for a rare disease such as EB clearly differs from the obvious demand generated by the sheer number of affected patients and the complexity of this multisystem disease. Currently, the German national action plan for rare diseases, “NAMSE” [7], is being implemented in certain states, with the establishment of level A and level B centres for coordination and treatment of rare diseases. Our results reflect the situation before its implementation. We suggest assessing the effects of this measure by another survey in the course of time. Excellent health care for rare skin diseases can only be provided if the specialised centres receive adequate appreciation, compensation and political support.

### Limitations

The number of responders is comparatively high and we estimate the probability of under-reporting of specialised EB centres as low. However, this survey relied on self-reporting of the departments addressed, which may lead to potential over-reporting of patient numbers or procedures. Health insurance data would be more objective, but are not publicly accessible and provide only partial coverage due to a large number of insurance companies within the German dual health-care system. For reasons of different health care systems in neighbouring countries, this survey was restricted to Germany.

## Conclusions

This survey of EB health care in Germany highlights the necessity and benefits of both disease-specific centres with concentrated expertise, and of smaller units with better reachability for patients’ everyday needs. This setting requires excellent networking and knowledge-transfer between physicians, other health care professionals as well as the patients. The findings of this EB survey may serve as a blueprint for other rare skin diseases.

## Methods

### Study population

All paediatric and dermatologic departments based in German hospitals and registered with the paediatric and dermatologic medical societies were addressed via email. For larger clinics (e.g. university hospitals) consisting of several paediatric departments dedicated to different subspecialties, each of these departments was addressed separately.

### Questionnaire

The questionnaire contained 29 questions on 7 topics: 1. General information about clinic type and location, 2. Characteristics of EB patients and the setting of care, 3. Diagnostics of EB, 4. The team of professionals involved in EB care, 5. Clinical and laboratory parameters for follow-up, 6. Participation in clinical trials, and 7. Personal experience regarding treatment of EB outside hospitals. The initial filter question “Do you treat individuals with epidermolysis bullosa?” determined whether the participant would undergo the complete interview; in case of a negative answer, the interview ended after general information on the respective clinic and its location had been obtained. The survey was conducted in German language. The questionnaire is available from the corresponding author upon request.

### Conducting the survey

The survey was designed and conducted using the SoSciSurvey platform, a freely available and non-commercial, cloud-based survey tool widely used in German-speaking countries (https://www.soscisurvey.de). Invitations were sent to participants by email. In case of non-reply, up to three reminder emails were sent. The survey period covered 9.5 weeks from 22/01/2018 to 31/03/2018.

### Data analysis

Data analysis was performed with descriptive statistics in R [25]. The additional R software package ggmap [26] was used for geographical displays and the package likert [27] was used to analyse and depict Likert scales.
